# RNA Therapeutic Options to Manage Aberrant Signaling Pathways in Hepatocellular Carcinoma: Dream or Reality?

**DOI:** 10.3389/fonc.2022.891812

**Published:** 2022-05-04

**Authors:** Kurt Sartorius, Samuel O. Antwi, Anil Chuturgoon, Lewis R. Roberts, Anna Kramvis

**Affiliations:** ^1^ Hepatitis Virus Diversity Research Unit, School of Internal Medicine, University of the Witwatersrand, Johannesburg, South Africa; ^2^ The Africa Hepatopancreatobiliary Cancer Consortium (AHPBCC), Mayo Clinic, Jacksonville, FL, United States; ^3^ Department of Surgery, KZN Kwazulu-Natal (UKZN) Gastrointestinal Cancer Research Centre, Durban, South Africa; ^4^ Division of Epidemiology, Department of Quantitative Health Sciences, Mayo Clinic, Jacksonville, FL, United States; ^5^ Discipline of Medical Biochemistry, School of Laboratory Medicine and Medical Sciences, College of Health Science, University of KwaZulu-Natal, Durban, South Africa; ^6^ Division of Gastroenterology and Hepatology, Mayo Clinic, Rochester, MN, United States

**Keywords:** small interfering RNA (siRNA), antisense oligonucleotide (ASO), aptamer, ribozyme, riboswitch, CRISPR/Cas9, RNAi strategies

## Abstract

Despite the early promise of RNA therapeutics as a magic bullet to modulate aberrant signaling in cancer, this field remains a work-in-progress. Nevertheless, RNA therapeutics is now a reality for the treatment of viral diseases (COVID-19) and offers great promise for cancer. This review paper specifically investigates RNAi as a therapeutic option for HCC and discusses a range of RNAi technology including anti-sense oligonucleotides (ASOs), Aptamers, small interfering RNA (siRNA), ribozymes, riboswitches and CRISPR/Cas9 technology. The use of these RNAi based interventions is specifically outlined in three primary strategies, namely, repressing angiogenesis, the suppression of cell proliferation and the promotion of apoptosis. We also discuss some of the inherent chemical and delivery problems, as well as targeting issues and immunogenic reaction to RNAi interventions.

## Introduction

Hepatocellular carcinoma (HCC) therapeutics remain an intractable global problem against a background of rising incidence and changing etiology (1). A wide range of novel HCC therapeutics have been developed and tested over the last two decades, including molecular targeted therapy, protein antibodies, targeted radionucleotide therapy, and epigenetic therapy. Other HCC therapies include immunotherapy, immune-cell therapy, differentiation strategy and RNA interference (RNAi) (2). Despite these advances a cure is yet to be developed and to date survival time has only been modestly extended (3). Central to the problem of HCC management is that this cancer remains (largely) asymptomatic until it is very advanced (4).

This review paper specifically investigates RNAi as a therapeutic option for HCC. RNAi therapeutics involves the deployment of RNA molecules in sequence-specific modulation to repress gene specific expression (5). In this paper we review the deployment of a range of RNAi technology including anti-sense oligonucleotides (ASO), Aptamers, small interfering RNA (siRNA), ribozymes, riboswitches and CRISPR/Cas9 technology for the management of HCC. The use of these RNA based interventions is specifically outlined in three primary strategies, namely, repressing angiogenesis, the suppression of cell proliferation and the promotion of apoptosis.

## RNAi Based Therapeutic Options in HCC

### Small Interfering RNA

**Figure 1:**
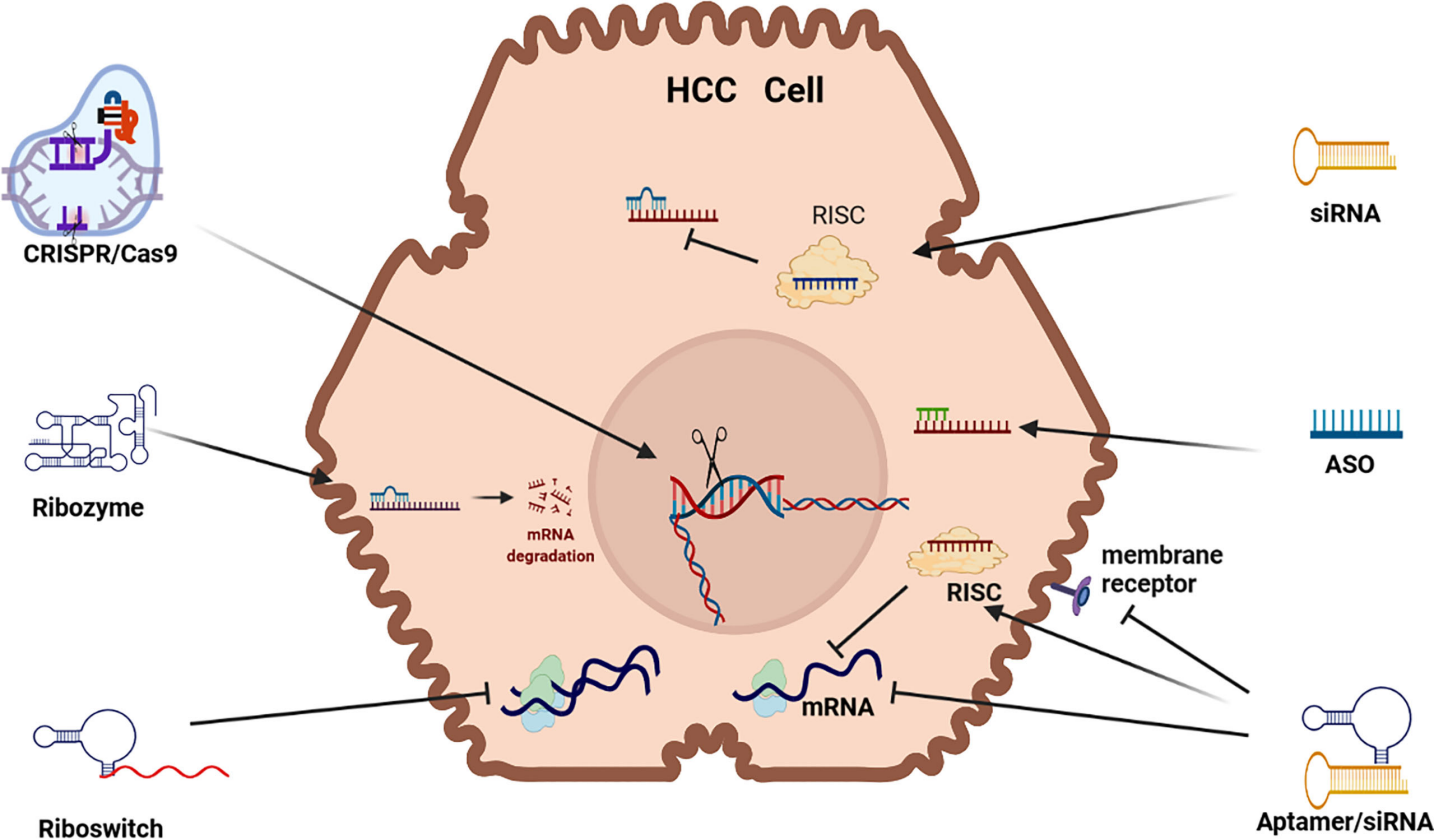
RNAi Therapeutic options for HCC. siRNA (small interfering RNA), ASO (antisense oligonucleotides, Aptamer, Riboswitch, Ribozyme, CRISPR/Cas9 (clustered regularly interspaced short palindromic repeats/Caspase-9) mRNA (messenger RNA) (mRNA) RISC (RNA-induced silencing complex).

Small interfering RNA (siRNA) are double stranded RNA (dsRNA) that load a guide strand into some form of Argonaut (AGO) based RNA-induced silencing complex (RISC) after separation from its passenger strand (see **Figure 1**). A RISC with its encapsulated RNA attach to a complementary strip of mRNA and cause repression or cleavage. In effect, the silencing process adopted by siRNA is identical to that of microRNA (miRNA) in nature which are endogenously synthesized by endonucleases (DICER) and the guide strand packaged in a specific AGO based RISC in the cytoplasm. In humans, naturally occurring siRNA have a specific target and their origin seems to be the result of responding to a pathogen encountered in the past. For therapeutic purposes, siRNA can be chemically produced and modified and delivered in a wide range of nanoparticles (NPs). To increase their efficacy, siRNA vectors have been developed to achieve longer term application including modifications like a cassette that includes RNA Pol 11 that can perpetuate the transcription of siRNA. Transcriptional gene silencing (TGS) can also be influenced by siRNA where the complementary bases create a complex that binds to DNA (6).

Exogenous siRNAs are dsRNA characterized by a 2nt overhang on the 3’ end of both strands that are delivered to an AGO based RISC that especially represses translation in the critical 2-8 position on the 5’ end of its target mRNA. Although siRNA therapeutics mostly deploys manufactured siRNA, endogenous siRNA can also be harnessed and deployed for RNAi by using a viral vector to express short hairpin RNA (shRNA). Alternatively, manufactured siRNA that are miRNA mimics can be utilized for RNAi. Manufactured siRNA-based therapeutics use siRNAs that have been chemically stabilized to increase their potency and reduce RNase degradation *in vivo* but challenges remain due to off-target effects, as well as unintended immunogenic reaction (7). The stabilization and delivery of siRNA is, therefore, a critical issue (8).

### Clustered Regularly Interspaced Palindromic Repeats/Caspase-9 (CRISPR/Cas9)

CRISPR/Cas9 uses CRISPR to guide Cas9 cleavage of targeted DNA sequences. This therapeutic application has been developed as a guide RNA/enzyme complex that can delete targeted RNA or DNA, as well as conduct precise DNA edits or inserts. CRISPR/Cas9 technology uses gRNA to guide and bind Cas9 cleavage to a its target DNA, however, CRISPR/Cas9 can also be used to re-engineer mutations, as well as ex vivo therapy to edit and then re-introduce edited cells *in vivo* (9). Cleavage of targeted DNA occurs by RNase 111. The CRISPR/Cas9 DNA gene replacement technology is developed in three steps: (i) insertion of the short sequence of the foreign DNA as a spacer in CRISPR array; (ii) transcription of the precursor cRNA (pre-cRNA) followed by its processing to generate individual cRNAs that comprise of a repeat and a spacer; and (iii) recognition of the target sequence by cRNAs and cleavage of the foreign nucleic acid by Caspase DNA nucleases at sites complementary to the cRNA spacer sequence. Repair of DNA following cleavage is activated by CRISPR-Cas9 gene editing that introduces a donor repair template with homology identical to the area of the dsDNA cleaved DNA. The CRISPR/Cas systems (1, 10, 11) can activate or repress gene expression (7).

### Antisense Oligonucleotides

Antisense oligonucleotides (ASOs) are short single stranded (ss) DNA/RNA that exist in nature as small RNA molecules. RNA based ASOs bind to a complementary nucleotide sequence site in mRNA causing splicing, degradation (R Nase H) or the repression of mRNA translation (6, 12) and DNA based ASOs form a DNA-RNA heteroduplex to precipitate RNases. Most ASO based drugs precipitate RNases but they can also repress translation, as well as be deployed to splice target DNA/RNA (7, 10). The chemistry and target sequence of ASOs determine their mechanism of action and the fate of the targeted RNA (13).

### RNA Aptamers

Aptamers are short, single-stranded DNA or RNA (ssDNA or ssRNA) molecules that can selectively bind to a specific target. Aptamers can fold into a multi-functional state to bind to a target DNA/RNA in a similar fashion to an antibody to suppress protein expression. Aptamers, often labeled as chemical antibodies, can bind to a wide range of molecules including toxins, viruses, proteins and peptides and its binding facility is not based on complementarity but rather its ability to fold into a 3-dimensional structure to enfold a target molecule in the same way as an antibody (2, 6). RNA aptamers can be used as a delivery vehicle for a wide range of chemical agents and are less expensive and illicit low immunogenicity response compared to antibodies. Aptamers, which have a unique niche in RNAi therapeutics, can be developed to bind to an intracellular or extracellular target, and they can be used to activate or repress target expression (14). RNA aptamers can also bind to cell surface proteins to block their function, for example they can bind to VEGFR to inhibit VEGF signaling (15). Alternatively, they can be used to deliver RNAi based cargo (siRNA, shRNA, miRNA) to the intracellular space where they are encapsulated by RISC and delivered to target mRNA (16).

### Ribozymes

Ribozymes are ribonucleic acid enzymes that play a catalytic silencing role to promote the cleaving of target mRNA by hybridizing with. This is a process where two ssRNA molecules combine into a single dsRNA molecule. There are three predominant forms of ribozyme, namely, hammerhead (HH), hepatitis delta virus (HDV) and hairpin (HP) ribozymes (7) but the most common ribozymes used in a therapeutic capacity take a “hammerhead” or “hairpin/paperclip” format (2). Ribozyme RNA molecules can catalyze specific biochemical reactions, as well as RNA splicing (trans-splicing ribozyme), and can be classified as genetic material (like DNA) or a catalyst. The most common activity of ribozymes includes cleavage/degradation of RNA/DNA but they can also play a role in linking peptide sequences (e.g. protein construction) within ribosomes.

Ribozymes can be directed to target the 5’ end of RNA to induce RNaseP and cleavage in a sequence specific manner. Liposome mediated ribozymes can be delivered exogenously or transcribed endogenously to cleave target mRNA and they can be used repeatedly because they are not consumed in the cleavage process. Ribozyme therapy can be used to target most non-coding RNA (ncRNA) including siRNA, microRNA (miRNA), long noncoding (lncRNA), piwi-interacting RNA (piRNA) and small nucleolar RNA (snoRNA) that all play a regulatory role in carcinogenesis (17, 18). Trans-cleaving ribozymes can target specific mRNA segments in the same way as a dedicated siRNA (19). Due to poor stability of fully-RNA based ribozymes, they are chemically stabilized for therapeutic deployment using some of the following modifications: 5′-PS backbone linkage, 2′-*O*-Me, 2′-deoxy-2′-*C*-allyl uridine, and terminal inverted 3′-3′ deoxyabasic nucleotides (20).

### Riboswitches

Riboswitches are a group of dynamic ncRNA motifs found upstream, in the 5′ untranslated region of mRNAs, where they regulate translation through binding of their cognate ligand to their aptamer domain (21). A riboswitch can be in an “on”/”off” state depending on the presence of a ligand. For example, in the absence of a ligand, the riboswitch folds to an “on” state and binds to the ribosome binding site (RBS) triggering translation initiation (RNA Polymerase). Conversely, in the presence of a ligand, the riboswitch folds to an “off” state to bar RBS binding and translation is blocked (7).

## RNAi Challenges and Delivery Options

### RNA Delivery Challenges

RNA can be delivered in their ‘naked’ state but they have a short circulation life *in vivo* due to speedy degradation *via* nuclease activity (22). Some of the problems of RNA-based therapeutics, therefore, include maintaining their stability and efficacy in sustained systemic delivery, as well as avoiding off-target effects. Other problems of RNA delivery include avoiding renal clearance, adverse immunogenic response, such as cytoplasmic RNA triggering IFN expression (2, 22, 23), and avoiding endosomal escape that can trigger unwanted TLR signaling (23). Additional problems arise because of the need to counter the negatively charged phosphate backbone of RNA, as well as its high molecular weight that reduce its stability and act as a barrier to cell entry and repress targeting ability (24). RNA based drugs are easily modified and their long term efficacy can be increased by the inclusion of replicative ability in the RNA based vesicle (6).

### Stabilization of RNA

To overcome delivery challenges, a range of chemical modifications can prolong RNA half-life without jeopardizing their biological activity. RNA can be stabilized by chemical modifications to both sense strands, as well as the 3’ and 5’ caps, as well as ribose modifications. Ribose modifications to the 2’-OH site are a common modification to stabilize RNA to be stabilized, including the following options, namely, 2’-Me, 2’-F and 2’-H. RNA molecules can also be stabilized by intramolecular linkages of 2’-Oxygen to 4’-carbon to create bridged nucleic acid (locked nucleic acid-LNA) molecules. Modifications to the RNA backbone can also increase nuclease resistance and involve substituting non bridging phosphate oxygen for sulfur, as well as boranophosphate substituted with the BH3 group (22). These modifications reduce off-target interference (25) and reduce immunogenicity response, mediated by TLR or PKR activation (26).

### Nanoparticle Delivery of RNA

A wide variety of nanoparticles (NPs) have been developed and tested over 40 years. Delivery of RNAi based drugs can utilize lipid-based (cholesterol and α-tocopherol) NPs, polymer-based (polyethylene-PEG) NPs, inorganic NPs and a range of exosome-mimetic nanovesicles (27, 28).

Lipid-based NPs (LNPs) include liposomes, micelles, emulsions and solid lipid nanoparticles (SLNPs). LNPs also include stable nucleic acid lipid particles (SNALPs) (29), lipidoid nanoparticles (LNs), and SLNPs (30). LNPs have a biochemical structure (phosphate/sugar group) that mimics a cell membrane thus allowing fusion with a target cell membrane and its transport RNA into a target cell (24). LNPs, for example, are extensively used to deliver siRNA and small activating ds RNA (saRNA) that target gene promoters (7).

Polymer-based NPs like Polyethylene (PEG) can be complexed with Polyvinylimine (PEI) to form a PEG-PEI based RNA conjugate that increases stability *in vivo*, as well as enhances the targeting of its RNA based cargo (31). PEG can also be complexed with Poly Lysine (PLL) and Poly Amidoamine (PAMAM) that consist of macromolecules designed to carry RNA. Natural polymers conjugated with RNA include chitosan, atelocollagen and Hyaluronic acid (HA) which, for example, readily binds to CD44 expressing cancer cells (32). Upon delivery in the cytoplasm, the RNAi cargo is released from the polymer-based NP by the cleavage of its di-sulfide linkages (22).

NPs can use a ligand to target delivery where these conjugates promote improved binding and reduced immunogenicity. Upon cell entry the NPs can be separated (cleaved) to facilitate RNA loading into an AGO based RISC. For example, a Ga1NAc-siRNA conjugate readily binds to ASGPR thus providing siRNA entry access to hepatocytes (8, 33). Antibodies are also efficient delivery vehicles for cell specific delivery because they are stable *in vivo* and also specific to a chosen target (34). The delivery of RNA can incorporate nano-emulsions (35) including cationic nano-emulsions which are highly potent, versatile delivery vehicles for siRNA to target pathogens or induce antibodies (31).

Inorganic-based RNA NPs consist of mesoporous silica nanoparticles, superparamagnetic nanoparticles, carbon nanotubes and gold nanoparticles and exosome-mimetic nanovesicles can also be used to transport RNAi cargo (28, 31). Viral vectors are highly effective delivery systems using genetically modified viruses to deliver genetic materials to the cells. Virus-based vectors have been proven to have several advantages in delivering the payload into cells *in vitro* and *in vivo*, including high efficacy by natural cell transduction, sustained gene expression, and long-term gene silencing. The use of an adenovirus vector encoding a siRNA, for example, can deliver RNA (36). Finally, aptamers can be modified for delivery of RNA and can be compared to antibodies with respect to their binding specificity. They are also stable, very small and easy to manufacture and can be combined with siRNA/CRISPR technology to target specific cell types (7, 31).

## RNAi Strategies

HBV-HCC pathways typically include aberrant expression in the retinoblastoma-tumor protein 53 (RB1-TP53) suppressor networks, the Wingless-related integration site/beta-catenin (WNT/β-Catenin) pathway, and the phosphoinositide 3-kinase/mitogen-activated protein kinase (PI3K/MAPK) and Janus kinase/signal transducer (JAK/STAT) pathways and transforming growth factor-beta (TGF-β) signaling (37). Typically, a range of growth factor receptors including VEGFR/IGFR/EGFR/PDGFR/FGFR and C-Met activate receptor tyrosine kinases in the P13/MAPK and RAS/RAF/MEK/ERK pathways (38). TGF-β signaling activates members of the Smad family of signal transducers (39) and activation of the WNT/B-Catenin pathway occurs *via* the binding of a WNT protein ligand to a Frizzled receptor (40). In addition, the binding of extracellular ligand (IL-6) leads to pathway activation of the JAK/STAT pathway (41).

**Figure 2:**
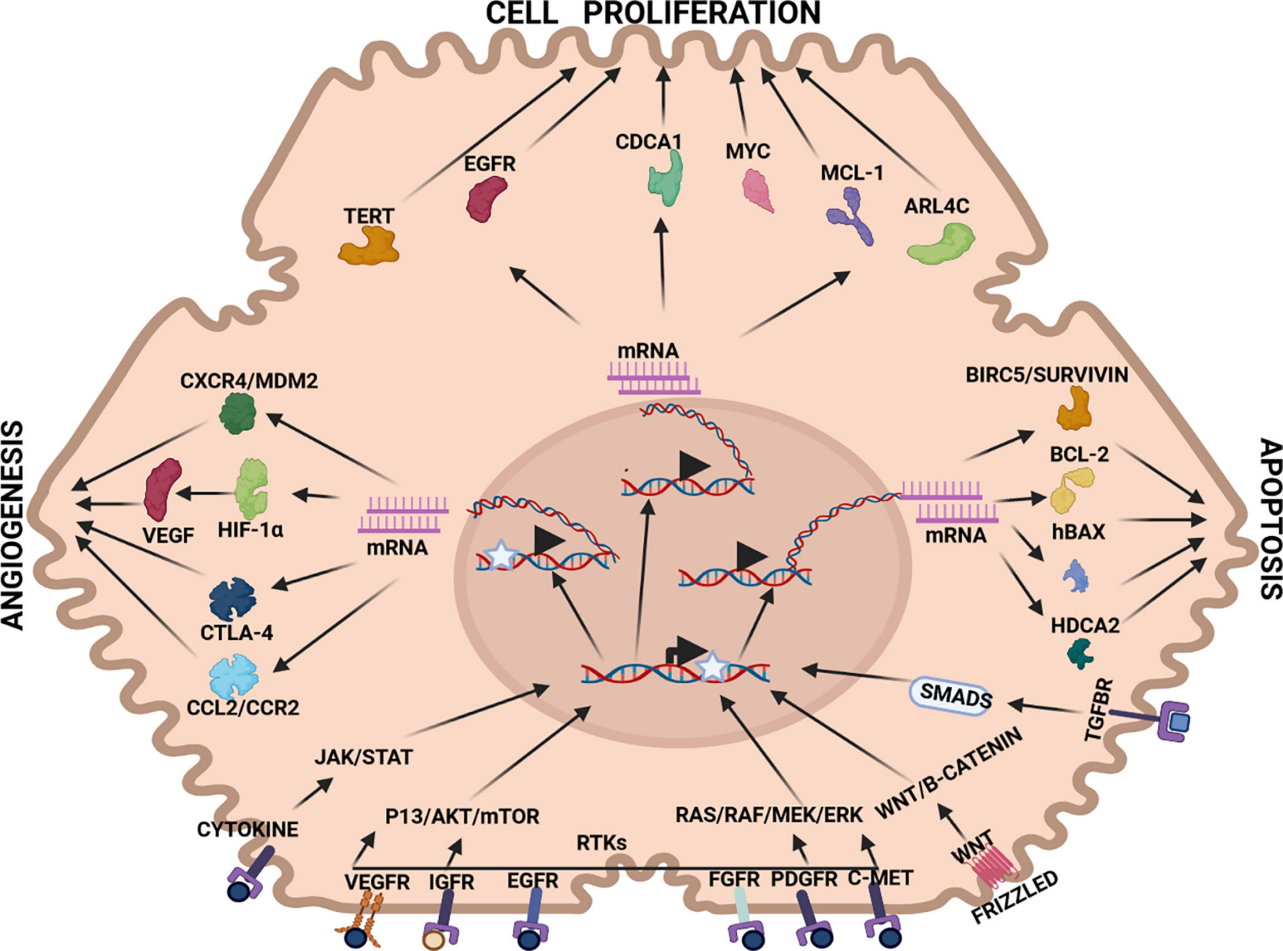
Therapeutic targets in HCC pathways: *Angiogenesis:* CXCR4/MDM2(chemokine receptor/murine double minute 2 protein), CTLA-4 (constitutively expressed in regulatory T cells), HIF-1α (hypoxia-inducible factor 1-alpha, a subunit of a heterodimeric transcription factor hypoxia-inducible factor 1 that is encoded by the HIF1A gene), VEGF (vascular endothelial growth factor, stimulates the formation of blood vessels), *Cell proliferation:* TERT (telomerase reverse transcriptase, a catalytic subunit of telomerase, EGFR (epidermal growth factor receptor), CDCA1 (Cell division cycle associated 1, an oncoantigen), MYC (transcription factor, proto-oncogene), MCL-1 (induced myeloid leukaemia cell differentiation protein), ARL4C (ADP-ribosylation factor-like 4C). *Apoptosis:* BIRC5/Survivin (baculoviral inhibitor of apoptosis repeat-containing 5 or survivin), BCL-2 (B-cell lymphoma 2, regulatory protein inducing or inhibiting apoptosis), hBAX (human Bcl-2-associated X protein, apoptosis regulator), HDCA2 (histone deacetylase 2, removed acetyl groups from lysine at the N’ terminal of core histones). *Receptors:* VEGFR (vascular endothelial growth factor receptor), IGFR (insulin-like growth factor receptor), EGFR (epithelial growth factor receptor), FGFR (fibroblast growth factor receptor), PDGFR (platelet-derived growth factor receptor), C-MET (tyrosine-protein kinase *Met* or hepatocyte growth factor receptor (HGFR)), WNT (Wingless and Int-1). *Pathways:* JAK/STAT (Janus kinase (*JAK*)-signal transducer and activator of transcription (*STAT*)), P13/AKT/mTOR (phosphatidylinositol-3-kinase (*PI3K*)/*Akt* and the mammalian target of rapamycin (*mTOR*) signalling pathway), RTKs (receptor tyrosine kinases), RAS/RAF/MEK/ERK (extracellular signal-regulated kinases), WNT/B-CATENIN (highly conserved pathway).

Three primary strategies are interrogated in this section, namely, anti-angiogenesis, anti-cell proliferation and pro-apoptosis. In each therapeutic strategy, aberrantly expressed mRNA in HCC pathogenesis have been identified and targeted by RNAi therapy (see **Figure 2**). For example, VEGF mRNA is often targeted in RNAi anti-angiogenesis therapy, MYC mRNA to repress cell proliferation and BIRC5/Survivin mRNA to induce a pro-apoptotic effect (see **Tables 1**–**3**).

**Table 1:** RNAi anti-angiogenesis therapeutics.

RNAi NP delivery method	Target mRNA	Outcome	Source
**siRNA**			
LCP-NP (siRNA)	ASGP-R/VEGF	Anti-angiogenic effect	(42)
LNP (siRNA and AMD3100)	CXCR4/SFF1α	Repressed angiogenesis	(43, 44)
LNP pH sensitive (siRNA + sorafenib)	VEGF	Reduced VEGF and induced apoptosis	(45)
Stabilized (siRNA)	VEGF	Repressed angiogenesis	(46, 47)
Gold NP (siRNA)	VEGF/P13K/AKT	Repress vascularization	(48)
PEG-SS-PLL NP (siRNA)	VEGF	Anti-angiogenic effect	(49)
LNP (shRNA)	ASGP-R/VEGF	Repressed angiogenesis	(50)
LNP (siRNA + sorafenib)	Survivin	Repressed growth/angiogenesis	(51)
UA-GT/PAH-Cit NP (siRNA)	ASGP-R/VEGF	Reduced vascularization	(52)
Stabilized siRNA transfection	FGF	Reduced hepatic endothelial cells	(53, 54)
Stabilized siRNA transfection	MDM2/PDGF-B	Reduced tumor growth	(55)
LNP (siRNA + Tremelimumab)	PD-L1/CTLA-4	Promote T/B cell response	(56, 57)
NP (siRNA)	C-Met/AKT	Reduced angiogenesis	(58, 59)
**CRISPR/Cas9**			
Crisp/Cas9 NP (HIF-1 α knockout)	HIF-1α DNA	Repressed angiogenesis and invasion	(60)
Crispr/Cas9 NP (gene-chemo-silica NP)	EGFR-p13K-Akt	Repress angiogenesis	(61)
**ASO**			
PS-LNA ASO NP	HIF-1α/VEGF	Reduced endothelial expression	(12)
Isoform-specific ASO	HIF-1α/HIF-2α	Repressed tumorigenesis	(62)
**Aptamer**			
RNA aptamer	CCL2/CCR2	Inhibited angiogenesis	(63, 64)
NP (peptide-mod-miR-195 + fasudil)	VEGF/ROCK2	Repress vasculogenic activity	(15)
**Ribozyme**			
Hairpin ribozyme	VEGF	Inhibit VEGF expression/tumor growth	(65)
Hammerhead ribozyme	VEGF/MMP-1	Target VEGF/MMP-1 angiogenesis	(66, 67)
**Riboswitch**			
AFP sensing Riboswitch	YAP/14-3-3σ	Repress angiogenesis	(68)

lipid-based nanoparticle (LNP), lipid/calcium/phosphate NP (LCP-NP), polyethylene glycol-poly(ϵ-benzyloxycarbonyl-l-lysine) NP (PEG-SS-PLL NP), urocanic acid-modified galactosylated trimethyl chitosan (UA-GT), poly(allylamine hydrochloride)-citraconic anhydride NP (UA-GT/PAH-Cit NP), phosphorothioate antisense oligonucleotides-locked nucleic acid NP (PS-LNA NP).

**Table 2:** RNAi anti-proliferation therapeutics.

RNAi NP delivery method	Target mRNA	Outcome	Source
**siRNA**			
Fa-PEG-g-PEI-SPION NP (siRNA)	TBLR1/SIK1	Inhibits growth/angiogenesis	(69, 70)
Lipid-polyplex NP (siRNA)	TERT/EGFR	Repressed cell proliferation	(71, 72)
LNP (siRNA)	b1 and av integrin	Repressed cell proliferation	(73)
Liposome-based NP (siRNA +AD3100)	RRM2	Inhibited growth	(74)
SNALP (siRNA)	CDCA1*	Reduced cell proliferation	(75, 76)
Stabilized siRNA transfection	CENP-A*	Inhibits HCC growth	(77)
LNP (siRNA)	MYC*	Inhibited tumor metabolism	(78)
LNP (siRNA)	PLK1*	Inhibits tumor growth	(79)
Stabilized siRNA	AQP3/HIPK3	Repressed proliferation	(11)
Stabilized siRNA	DLGAP5	inhibited cell proliferation	(80)
Stabilized siRNA	FoxM1/MMP-2/uPA	repressed proliferation	(81)
Stabilized siRNA	VEGF/KSP	anti-proliferation/pro-apoptosis	(82)
Stabilized siRNA	NET-1/EMS1/VEGF	anti-proliferation/pro-apoptotic	(83)
**CRISPR/Cas9**			
Crisp/Cas9 NP (NSD1 knockout)	NSD1	Repressed H3/WNT signaling	(84)
Crisp/Cas9 NP (TET knockout)	TET (1-3), SRY/UTY	Reduces cell proliferation	(85)
CRISPR/Cas9 NP (gene-chemo-silica)	PD-1	Enhanced T cell response	(86)
CRISPR/CAS9 (gene disruption)	PTEN/P53	Demonstrates TS role	(87)
**ASO**			
Stabilized ASO	MCL-1	Repressed MCL-1 expression	(88)
2-O-methyl modified ASO	STAT3	Inhibits HCC growth	(89)
Lipid ASO (PTO backbone)	MYC	Impedes tumorigenesis	(90)
Isoform-specific ASO	HIF-1α/HIF-2α	Repressed tumorigenesis	(62)
PTO backbone ASO	ARL4C	Inhibited proliferation	(91)
**Aptamer**			
RNA aptamer (guided by AFP)	C-jun/C-fos/nucleolin	Reduced	(92)
ssDNA aptamer	GAL-14	expression/proliferation	(93)
**Ribozyme**			
Trans-splicing ribozyme	hTERT	GAL-14 increase/reduced	(6)
Trans-splicing ribozyme	AFP	growth	(94)
**Riboswitch**			
RBP/Riboswitch	MS2	Inhibits tumorigenesis	(95)

folate- polyethylene glycol-grafted polyethylenimine and superparamagnetic iron oxide NP (Fa-PEG-g-PEI-SPION NP), Stable nucleic acid lipo-particle (SNALP), Lipid-based NP (LNP), RNA-Binding protein (RBP) phosphorothioate modified backbone (PTO) *clinical trial stage.

**Table 3:** RNAi pro-apoptotic therapeutics.

RNAi NP delivery method	Target mRNA	Outcome	Source
**siRNA**			
PEI NP (shRNA+ sorafenib)	Survivin	Inhibited growth, angiogenesis	(51)
RGD-PEG-*g*-PEI-SPION NP (siRNA)	Survivin	Promotes apoptosis	(96)
NP (siRNA-miR-122)	PKM2/HDAC-8	Induces apoptosis	(97–99)
SNALP (siRNA)	HDCA2*	p27/p53-led apoptosis/BCL-2	
NP (siRNA +cisplatin)	hTERT	down	(100, 101)
NP (siRNA)	CYCLIN-E	G2/M/S control -led apoptosis	(102)
NP (siRNA)	CXCL1	Induces apoptosis	(103)
NP (siRNA)	PDGFR-ß	Induces apoptosis and cell	(104)
Polymeric NP	VEGF/survivin*	growth	(105)
Polymeric NP	VEGF	Represses cell survival	(106)
			(107)
**CRISPR/Cas9**			
Crisp/Cas9 NP (insert HSV1)	HSV1 DNA	Induce apoptosis	(108)
Crisp/Cas9 NP (targeted PD-1)	PD-1	Induce apoptosis	(86)
**ASO**			
Chemically stabilized ASO	TERT	Promoted T-cell response	(109)
**Aptamer**			
ssDNA aptamer (GT repetition)	eEF1A	Targets cell survival/apoptosis	(110)
**Ribozyme**			
Trans-splicing ribozyme/cleave/replace	Mutant TP53	Replace TP53 promote apoptosis	(111)
Trans-splicing ribozyme	hTERT	Replacement of TERT RNA	(112–114)
Trans-splicing ribozyme	AFP	Promotes cell suicide	(94)
Trans-splicing ribozyme	VEGF/MMP-1	Promotes autophagy	(67)
**Riboswitch**			
RBP/Riboswitch	cNOT7/BCl-2/hBAX	BCL-2 up/hBAX down/apoptosis	(95)
(RBP)/riboswitch	YAP/14-3-3σ	Induces cell degradation	(68)

Polyethylenimine NP (PEI NP), tripeptide arginine glycine aspartic acid (RGD)-modified non-viral vector, polyethylene glycol-grafted polyethylenimine functionalized with superparamagnetic iron oxide NP (RGD-PEG-g-PEI-SPION NP), Stable nucleic acid lipo-particle (SNALP). RNA-binding protein (RBP) *clinical trial stage.

### Anti-Angiogenesis RNAi Strategies

**Figure 3:**
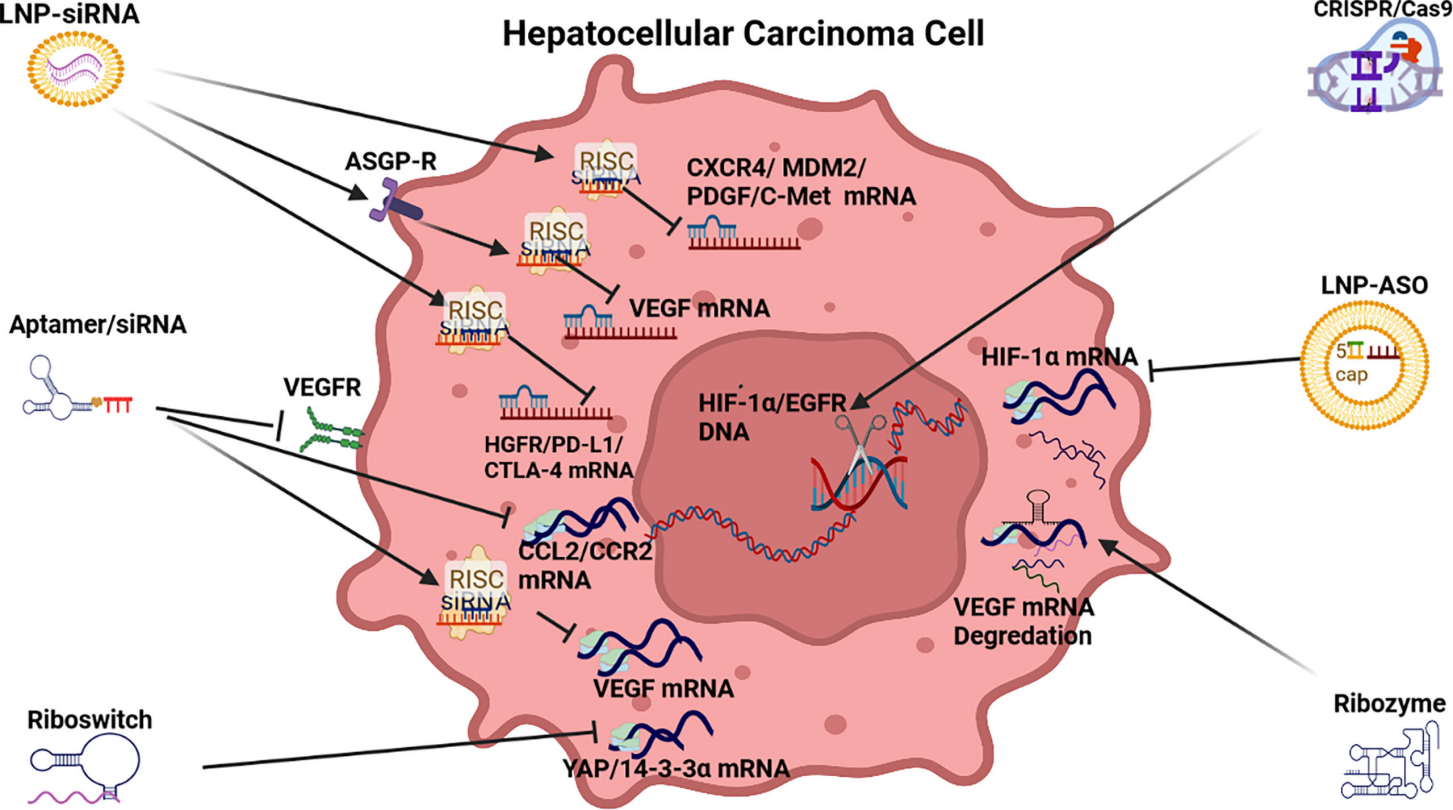
RNAi anti-angiogenic strategies. LNP-siRNA (Lipid nanoparticles for targeted small interfering RNA delivery), CRISPR/Cas9 V (clustered regularly interspaced short palindromic repeats/CRISPR-associated protein 9), LNP-ASO (lipid nanoparticle associated antisense oligonucleotide), siRNA (small interfering RNA). VEGFR (vascular endothelial growth factor receptor), ASGPR (asialoglycoprotein receptor), RISC (RNA-induced silencing complex), HIF-1α (hypoxia-inducible factor 1-alpha, a subunit of a heterodimeric transcription factor hypoxia-inducible factor 1 that is encoded by the HIF1A gene), CCL2/CCR2 (chemokine *CCL2* and its main receptor *CCR2*), HGFR/PD-L1/CTLA-4 (human growth factor receptor/programmed death-ligand 1/constitutively expressed in regulatory T cells), CXCR4/MDM2/PDGF/C-Met [chemokine receptor/murine double minute 2 protein/platelet-derived growth factor/tyrosine-protein kinase *Met* or hepatocyte growth factor receptor (HGFR)].

HCC pathogenesis including hypervascularity and marked vascular abnormalities are common feature of disease progression in HCC (115). Vascular abnormalities encompass the arterialization of tumor blood supply, as well as sinusoid capillarization that is promoted in the presence of hypoxia expressed HIF-1α (116). At a molecular level, the drivers of angiogenesis override their inhibitors (117). The drivers of angiogenesis include a range of growth factors (GFs) that include the vascular endothelial growth factor and its receptors (VEGF/VEGF-R), fibroblast growth factors and their receptors (FGF/FGF-R) and platelet derived growth factors and their receptors (PDGF/PDGF-R) (see **Figure 3**). FGFs, in particular, play an important role in angiogenesis and cross-signaling between FGF-2 and VEGF-A occurs early in HCC pathogenesis inducing neovascularization, increased capillarization of sinusoids and tumor growth. FGF expression also promotes angiogenesis by modulating integrin related regulation of endothelial cells in the microenvironment (117, 118). The FGF1, FGF2, FGF4, and FGF8 subfamilies are the most frequently studied FGFs in the angiogenic processes of HCC and FGF2 (in particular) is a characteristic factor in angiogenesis. FGF2 mainly targets FGFR1 as its receptor to mediate angiogenic multiple angiogenic stages (117, 119).

Other growth factors driving angiogenesis are hepatocyte growth factor (HGF) and its receptor c-Met, angiopoietins (ANG1/2 and Tie2) and endoglin (CD105) (117, 120–123). Endoglin (CD105) is a type-1 integral transmembrane glycoprotein and coreceptor for transforming growth factor-β (TGF-β) ligands that promotes angiogenesis in HCC by participating in the neovascularization process (124). Typically, the drivers of angiogenesis induce endothelial cell tyrosine kinases in the P13/Akt/mTOR cancer pathway. Conversely, angiogenesis inhibitors include angiostatin, endostatin and thrombospondin-1 (125).

The development of RNAi drugs to repress angiogenesis in HCC has been promoted by the moderate efficacy of the Sorafenib family of drugs. RNAi therapeutics to repress angiogenesis include the deployment of siRNA, aptamers, riboswitches, ribozymes, ASOs and CRISPR/Cas9 (See **Table 1** and **Figure 2**).

**Small interfering RNA:** This branch of therapeutics has largely focused on siRNA repression of the drivers of angiogenesis in HCC including VEGF, FGF, PDGF, HGF, angiopoietins (ANG1/2), endoglin (CD105) and their respective receptors. A preponderance of siRNA therapeutic models targeting angiogenesis in HCC have targeted vascular endothelial growth factor (VEGF) expression (117) possibly because Sorafenib and its descendants, FDA approved antiangiogenic agents targeting VEGF, have had modest efficacy (126). RNAi based VEGF siRNAs, delivered by a range of organic and inorganic nanoparticles (NPs), have proved effective to repress VEGF mRNA translation to demonstrate an anti-angiogenic effect on HCC pathogenesis both *in vitro* and *in vivo* (see **Table 1**).

Innovative examples of VEGF delivery using lipid based NPs include a VEGF-siRNA-sorafenib co-loaded pH sensitive liposomes that exhibited enhanced VEGF downregulation and anti-angiogenic effect, as well as induced apoptosis (45). In another model, AMD3100, a CXCR4 antagonist associated with HCC progression (43), as well as VEGF siRNA was delivered by an LNP demonstrated the ability to repress angiogenesis and tumor growth in HCC (44). AMD3100 is a molecule that can block the SDF1α/CXCR4 axis and sensitizes HCC to sorafenib treatment (127). Some lipid-based NPs (LNPs) have used the ASGP-R to gain cell entry and guide siRNA or shRNA delivery to repress VEGF expression. This form of cell entry that is facilitated by the recognition of galactose receptors, also demonstrated effective endosome escape (42, 50, 52). Another innovative LNP included both siRNA and a drug (Tremelimumab) to repress angiogenesis *via* the PD-L1/CTLA-4 pathway (56) while LNP-siRNA modulation of PD-L1/PD-1 acted as an immunoinhibitory receptor to express T/B cells to overcome the tolerance to angiogenic activity in the liver (128).

VEGF delivery using an inorganic gold NP containing VEGF siRNA repressed tumor revascularization in HCC *in vitro* and *in vivo* (48). In another case, a polymer NP complex was used to transport VEGF siRNA and demonstrated a marked anti-angiogenic effect *in vitro* and *in vivo* (49). An alternate siRNA design involved methylation of VEGF siRNA ends to stabilize them *in vivo* and this therapy reduced HCC induced angiogenesis through inactivation of VEGF/PI3K/AKT signaling pathway (46). In another example, stabilized VEGF siRNA significantly reduced VEGF expression and resulted in the knockdown of endothelial cell production *in vitro* and reduced tumor size *in vivo* (47).

FGF targeted siRNA models have been largely anchored in a series of *in vitro* studies and there is little evidence yet that siRNA-based therapeutics have progressed to *in vivo* applications. For example, transfections of FGF8, FGF19, FGFR4 with siRNA in a range of HCC cell-lines significantly repressed the proliferation and tube formation of hepatic endothelial cells (53, 54). The HGF/c-Met axis is involved in cell proliferation, movement, differentiation, invasion, angiogenesis, and apoptosis by activating multiple downstream signaling pathways (129). Additionally, C-Met (HGF-R) may induce VEGF-A expression to enhance angiogenesis (130). A wide range of siRNA experiments have also targeted the HGFR induced tyrosine kinases *in vitro* and *in vivo* (58, 59, 129, 131, 132).

Similarly, PDGFs are secreted growth factors closely related to VEGF and are important in HCC-induced angiogenesis because they activate tyrosine kinases contributing to the upregulation of VEGF and recruitment of perivascular cells (133). Stabilized Mdm2 siRNA or PDGF-B siRNA, transfected into an Hep3B cell line plasmid, demonstrated angiogenic effects *in vivo*, as well suppressed tumor growth (55). Stabilized siRNA also reported good efficacy in a murine mammary adenocarcinoma experiment to target endoglin which as a receptor for TGF-β signaling (134). Finally a polymer NP complex transporting shRNA and sorafenib and surviving shRNA inhibited tumor growth and angiogenesis for resistant HCC (51).

**CRISPR/Cas9:** The use of CRISPR/Cas9 therapy in HCC offers an exciting new option to manage pathogenesis. To date examples of this therapy in HCC include an HIF-1α knockout in a xenograft model to repress angiogenesis and invasion (60). HIF-1α is a key transcription factor involved in the hypoxic response of cancer cells. It activates transcription of genes responsible for angiogenesis, glucose metabolism, proliferation, invasion and metastasis in HCC (135). In another *in vivo* HCC study, a CRISPR/Cas9 system was deployed for gene–chemo-combination therapy that used a silica nanoparticle to initiate CRISPR/Cas9 editing of EGFR, as well as the co-delivery of sorafenib to modulate EGFR-p13K-Akt expression and repress angiogenesis (61).

**ASOs:** There has been a limited application of ASO-based RNAi for this strategy. One example of an anti-angiogenic ASO repressed HIF-1α led VEGF expression (12).

**Aptamers:** Although the use of aptamers in RNAi is less evident than other anti-angiogenic strategies in HCC, there are some examples. Aptamers targeting of CCL2/CCR2 and CCR2 expression using small-molecule antagonists, neutralizing antibodies, or RNA aptamer-based inhibitors have demonstrated efficacy to repress angiogenesis in subcutaneous HCC xenografts and endogenous liver tumors (63, 64). In another study, an aptamer-functionalized peptide (H3CR5C) was deployed to co deliver fasudil and miRNA-195 in a nanoparticle to inhibit VEGF expression and suppress vasculogenic activity by repressing ROCK2 (15).

**Ribozymes**: Ribozyme therapeutics in HCC used an anti-VEGF hairpin ribozyme to effectively inhibit VEGF expression and the tumor growth *in vitro* and *in vivo* (65). In another *in vivo* study, an anti-VEGF-A hammerhead type ribozymes suppressed not only VEGF mRNA but also VEGF protein (66, 67).

**Riboswitch**: In one HCC study a riboswitch that could sense AFP triggered the promotion of YAP induced 14-3-3σ expression to repress angiogenesis and promote degradation (68).

### Anti-Cell Proliferation RNAi Strategies

**Figure 4:**
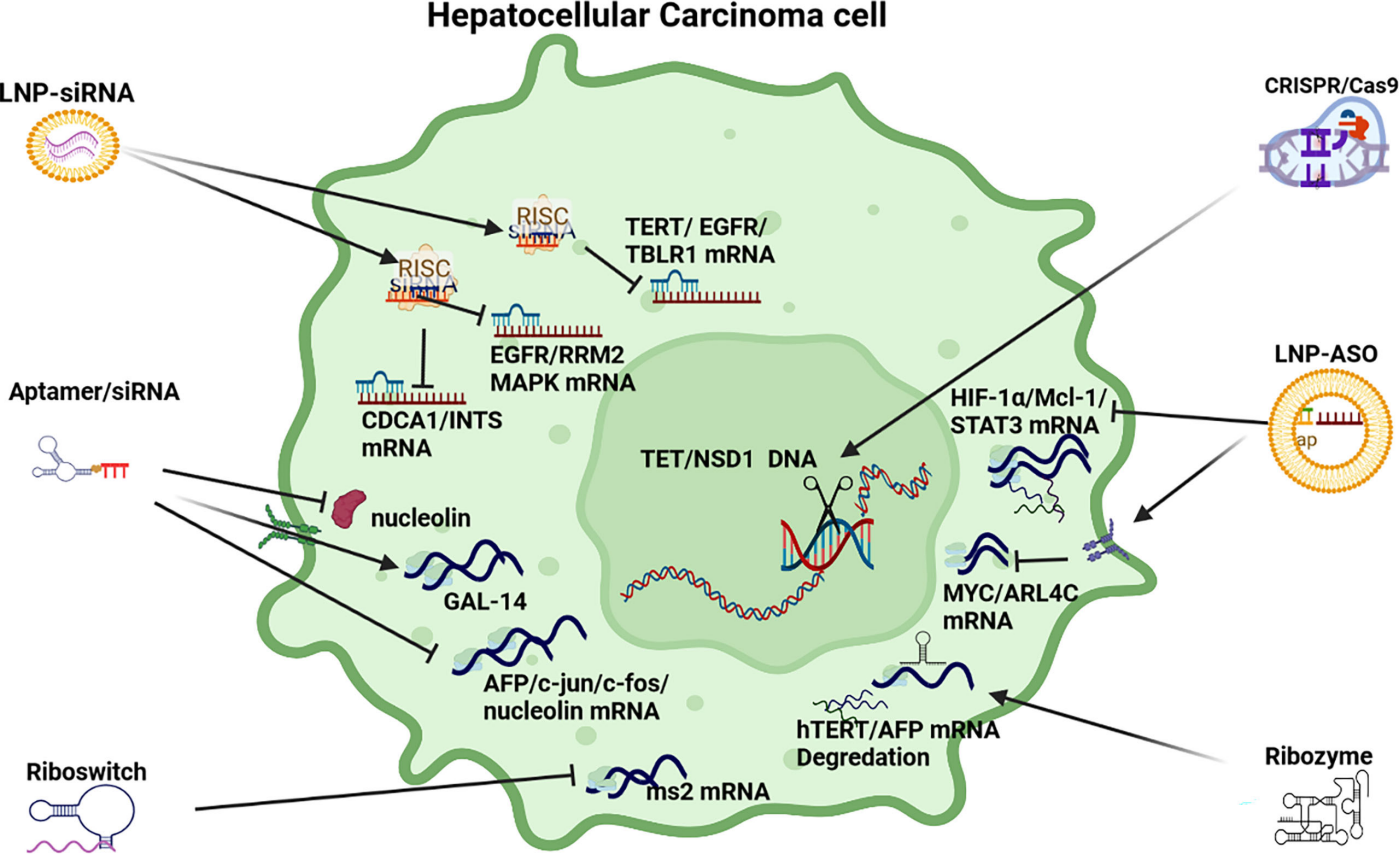
RNAi anti-cell proliferation strategies. LNP-siRNA (Lipid nanoparticles for targeted small interfering RNA delivery), CRISPR/Cas9 V (clustered regularly interspaced short palindromic repeats/CRISPR-associated protein 9), LNP-ASO (lipid nanoparticle associated antisense oligonucleotide), siRNA (small interfering RNA). HIF-1α/Mcl-1/STAT3 (hypoxia-inducible factor 1-alpha, a subunit of a heterodimeric transcription factor hypoxia-inducible factor 1 that is encoded by the HIF1A gene/induced myeloid leukaemia cell differentiation protein gene/signal transducer and activator of transcription 3), MYC/ARL4C (transcription factor, proto-oncogene/ADP-ribosylation factor-like 4C), hTERT/AFP (human telomerase reverse transcriptase/alpha fetoprotein), AFP/c-Jun/c-fos/nucleolin (transcription factors), GAL-14 (galectin 14), CDCA1/INTS (Cell division cycle associated 1, an oncoantigen), EGFR/RRM2/MAPK (epidermal growth factor receptor/ribonucleoside-diphosphate reductase subunit M2/mitogen-activated protein kinase), TERT/EGFR/TBLR1 [telomerase reverse transcriptase, a catalytic subunit of telomerase/transducin (beta)-like 1 X-linked receptor 1].

**Cell Proliferation Pathways in HCC**: Several signaling pathways in HCC influence cell proliferation in HCC pathogenesis including the TGF-β, Wnt/B-catenin, Hedgehog (Hh), Notch, JAK/STAT and Hippo pathways (see **Figure 4**). TGF-β upregulates the expression of Snail, downregulates E-cadherin to promote epithelial to mesenchymal transition (EMT) and metastasis. The TGF-β signaling pathway also influences the preservation of the cancer stem cell (CSC) subpopulation and cell proliferation (136). In HCC pathogenesis, aberrant Wnt/β-catenin signaling is often induced by *CTNNB1* mutations leading to the accumulation of β-catenin in the nucleus and cytoplasm to promote vascular invasion, cell proliferation, and poorly differentiated tumors (40, 137), as well as cross-talk with hypoxia signaling pathways (138).

The Hedgehog (Hh) pathway also plays an important role in cell proliferation and invasion (139), as well as in EMT (140) while there is growing evidence that supports the critical role of Notch signaling in HCC in the regulation of the tumor microenvironment, tumorigenesis, progression, angiogenesis, invasion and metastasis (141). Furthermore there is evidence that a Notch1-Snail1-E-cadherin type association affects invasion and metastasis in HCC pathogenesis (142). The JAK/STAT signaling pathway plays important roles in many cellular functions, including cell proliferation activated by the transcription CCND1, BIRC5 and Mcl-1 (143) and the repression of Hippo signaling in HCC pathogenesis *via* Hippo kinase (Mst1 and Mst2) also plays a role in the promotion of proliferation (144). Finally, the MAPK/ERK signaling pathway is activated in more than 50% of human HCC cases (145) by a range of growth factor receptors in HCC including VEGF-R, EGF-R, FGF-R, PDGF-R and C-MET that interlink with angiogenic expression (146).

**siRNA**: RNAi based therapeutics to modulate cell proliferation in HCC pathogenesis has deployed a range of polymer and lipid-based NPs, Stable nucleic acid lipo-particles (SNALPs), and chemically stabilized siRNA (see **Table 2**). For example, a polymer-based NP delivering TBLR1 siRNA inhibited growth and angiogenesis in HCC in both *in vitro* and *in vivo* applications. TBLR1 acts as a key oncogene in HCC to promote cell proliferation and angiogenesis in the WNT/B-Catenin pathway (69). In another example, a lipid-polyplex based NP delivering TERT and EGFR shRNA effectively repressed cell proliferation (71). EGFR is (often) highly expressed in HCC and is associated with cell proliferation and an aggressive phenotype that is prone to metastasis (147) Upregulated TERT as a result of mutations in the promoter region are also a frequent event in HCC and are significantly linked to disease progression (72). Another anti-cell proliferation started used LNPs injected into HCC mouse models to target integrin and CDCA1 expression (73). In support of this approach, integrins are found in HCC tissue but not in normal liver tissue and are associated with proliferation and invasion (148). Another SNALP based NP targeting CDCA1 also demonstrated reduced cell proliferation and pro-apoptotic activation as well as linkages with immune response (75, 76).

In another study a liposome complex NP model delivering a siRNA cargo plus Adriamycin inhibited growth by repressing RRM2 in EGFR expressing HCC (74). Interestingly, the elevated expression of RRM2 signifies a poor prognosis in HCC (149). Overexpression of CENP-A is frequently observed in HCC and a stabilized siRNA plasmid model targeting CENP-A reduced proliferation *via* repression of gene expression promoting cell cycle and inhibiting apoptosis (77). In a phase 1 trial, a Dicer-substrate small interfering RNA (DsiRNA) targeting MYC, exhibited some potential to repress cell proliferation (78). In HCC PLK1 expression, which is associated with cell cycle, is often elevated and a phase 2 trial using an LNP siRNA model is currently in progress to determine its use as an HCC drug to modulate proliferation/apoptosis (79).

Other *In vivo* based studies deploying stabilized siRNA have yielded positive results to illustrate the potential of RNAi. For example, stabilized siRNA targeting HIPK3 mRNA regulated tumorigenesis *via* the miR-124-AQP3 axis (11) while another stabilized siRNA model repressed DLGAP5 repression to suppress cell growth, migration and colony formation *in vitro* (80). Down-regulation of FoxM1 by stabilized siRNA also reduced the expression of matrix metalloproteinase-2 (MMP-2) and urokinase plasminogen activator (uPA) resulting in reduced cell proliferation in HCC cell lines (81). Silencing of VEGF and KSP mRNA by siRNA inhibited cell proliferation, migration, invasion and induced apoptosis in Hep3B cell-line experiments and it was hypothesized that this could be explained by the significant downregulation of Cyclin D1, Bcl-2 and Survivin (82). In a more recent study, a multi-target siRNA model inhibited NET-1, EMS1 and VEGF mRNA in HCC cells resulting in a significant reduction in proliferation, migration, invasion, angiogenesis and induced apoptosis in HCC cells (83).

**CRISPR/Cas9**: In a novel *in vivo* application of this therapy in HCC, CRISPR/Cas9 was used to test the hypothesis that deactivated PTEN/p53 genes in HCC promote carcinogenesis (87). In another application of CRISPR/Cas9 technology the expression of five oncogenes (*Tet1*, *2*, *3*, *Sry*, *Uty -* 8 alleles) was disrupted to reduce cell proliferation and highlight the key role of methylation of DNA in HCC (85). In addition, CRISPR/Cas9 therapy has been used to promote immunotherapy by modifying PD-1 in immune cells to promote T-cell response (86). In another example, a NSD1 knockout cell line was constructed by a CRISPR/Cas9 editing system to demonstrate its potential therapeutic application to HCC cell proliferation, migration and invasion as a result of NSD1 positive role in the expression of histone H3, Wnt10b and Wnt/β-catenin signaling (84).

**ASOs**: ASOs have been widely deployed in HCC to suppress oncogene expression that results in promoting cell proliferation. Oncogenes suppressed by a range of stabilized ASOs include *Mcl-1, HIF-1α*, *MYC* and *STAT3* (62, 88–90). MYC mRNA expression in HCC has also been silenced by siRNA based on a combination of 13 miRNA to suppress aberrant signaling promoting cell proliferation (2) and an MYC ASO model suppressed cell proliferation in mouse and human transgenic models (90). HCC cell proliferation was also repressed in another *in vitro* ASO based study which targeted ARL4C expression which is often highly expressed in HCC (91).

**Aptamer:** In 2012, a first report demonstrated that an RNA aptamer was able to detect cancer cells and inhibit proliferation in an alpha-fetoprotein (AFP) expressing HCC cell-line *via* the suppression of oncogene expression in *c-jun* and *c-fos*. This aptamer can also targeted nucleolin (92). In a later study a modified aptamer reduced cell proliferation by the upregulation of GAL-14 expression which is linked to immune response (93).

**Ribozyme**: In one study it was demonstrated that a trans-splicing ribozyme-mediated replacement of HCC-associated specific RNAs could target and replace AFP mRNA, which becomes increasingly expressed in HCC pathogenesis (94). In another study, a trans-splicing ribozyme was deployed to target hTERT expression which is elevated in HCC pathogenesis and promotes cell proliferation and survival (6).

**Riboswitch:** In one study deploying this therapy, a chimeric RNA-binding protein-based killing switch targeting MS2 to reduce cell priliferation in HCC (95).

### Pro-Apoptotic RNAi Strategies

Apoptosis pathways become increasingly dysregulated in HCC pathogenesis. Mutation od the p53 tumor suppressor gene is a common feature, as well as the disruption of the TGF-β family of cytokines that play a prominent role in apoptosis (150, 151). Other alterations include mutations in the Hedgehog pathway to promote anti-apoptotic resistance (152), as well as dysregulation of the death receptor pathways which include Fas-mediated caspase induced apoptosis (153). The upregulation of many anti-apoptotic proteins is also a common feature including NF-κB, Bcl-2 and Bcl-X_L_, as well as the downregulation of pro-apoptotic expression by *BID, BAX* and *BCL-Xs* (154). Tumor necrosis factor (TNF) common feature in HCC (155) alongside upregulated IGF and IGF-1 signaling pathways which can activate STAT signaling (156). Finally, the repression of many tumor suppressors like *SOCS-1/3* can also promote JAK/STAT signaling (157) while *PTEN* dysregulation promotes P13/MAPK/Akt signaling (158).

**Figure 5:**
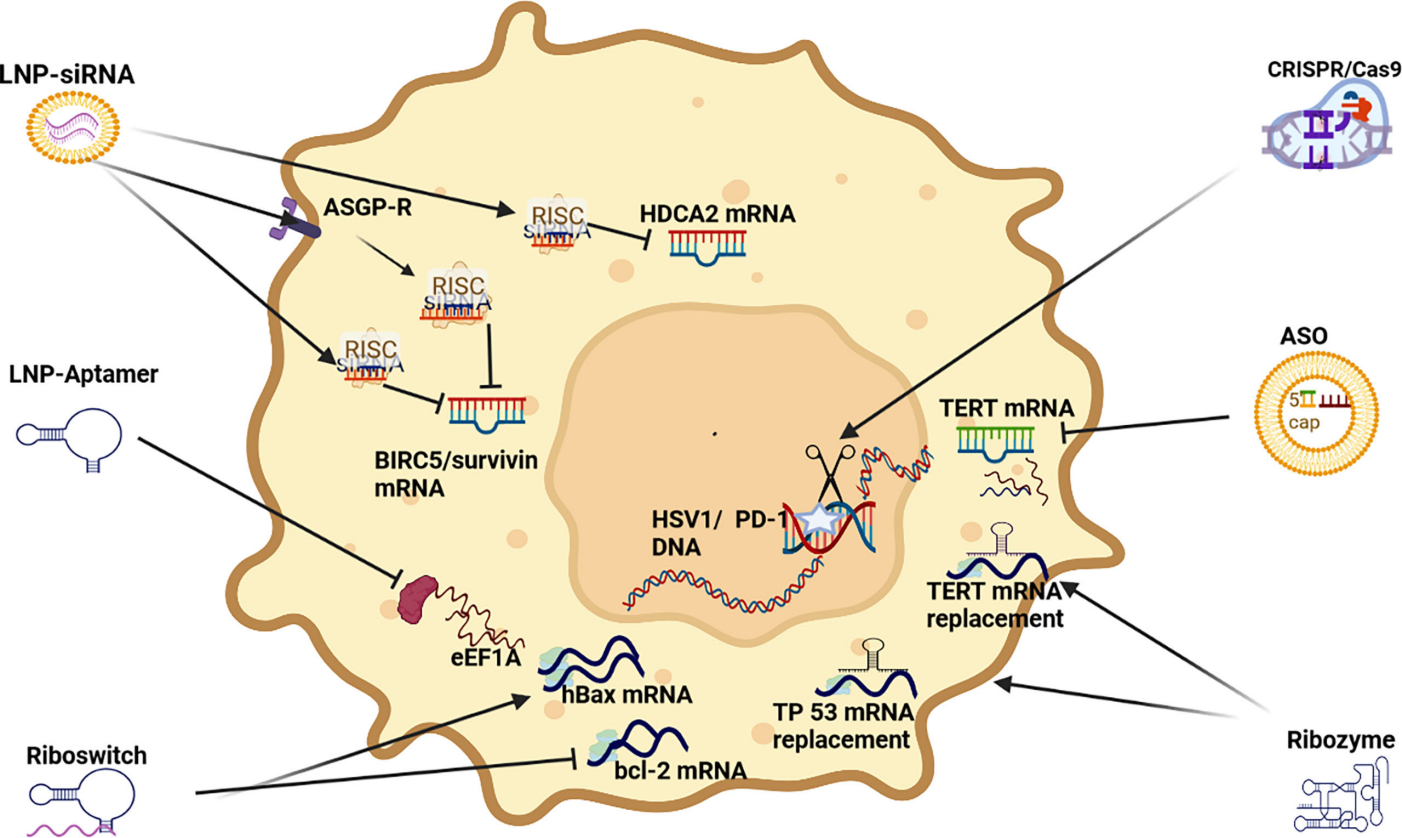
RNAi apoptosis strategies LNP-siRNA (Lipid nanoparticles for targeted small interfering RNA delivery), CRISPR/Cas9 V (clustered regularly interspaced short palindromic repeats/CRISPR-associated protein 9), LNP-ASO (lipid nanoparticle associated antisense oligonucleotide), siRNA (small interfering RNA); HIF-1α (hypoxia-inducible factor 1-alpha, a subunit of a heterodimeric transcription factor hypoxia-inducible factor 1 that is encoded by the HIF1A gene), TERT (telomerase reverse transcriptase), hBAX (human Bcl-2-associated X protein, apoptosis regulator), eEF1A (Eukaryotic translation elongation factors 1 alpha), BIRC5/Survivin (baculoviral inhibitor of apoptosis repeat-containing 5 or survivin), RISC (RNA-induced silencing complex), ASGP-R (asialoglycoprotein receptor), HDCA2 (histone deacetylase 2, deacetylates lysine of N’ terminal of core histones).

**siRNA**: Multiple RNAi pro-apoptotic siRNA strategies have been developed and tested, especially those targeting BIRC5/survivin. BIRC5/Survivin is often highly expressed in HCC and has been linked to cell proliferation, inhibiting apoptosis and promoting stromal angiogenesis (159) (see **Figure 5**). A co-polymer complex based NP has been operationalized with superparamagnetic oxide to deliver Survivin siRNA to successfully induce apoptosis (96). In another study targeting Survivin, a polymer based NP was used to deliver survivin shRNA and sorafenib to repress tumor growth and inhibit angiogenesis (51). Survivin is a recently described inhibitor of apoptosis and its overexpression translates into a decrease in the G_0_/G_1_ phase and an increase in the S phase in cell cycle controls resulting in the activation of cell cycle network (160–162).

A stabilized siRNA (based on miR-122) model targeting PKM2, induced apoptosis and growth arrest by downregulating PKM2 in HCC *in vitro* and *in vivo* (97). In another stabilized siRNA model both HDAC8 and PKM2 mRNA expression was reduced to promote pro-apoptotic effects. Interestingly, HDAC8 caused PKM2 to translocate to the nucleus and synergize with *β-catenin* transcription to upregulate the expression of cyclin D1 (98). SNALP based NPs have also been used to transport both HDAC1 and HDAC2 siRNA to induce apoptosis by upregulating p57/p27 expression, as well as downregulating bcl-2 expression (100, 101) which is associated with a pro-survival effect in HCC (163). Another stabilized siRNA model demonstrated the synergistic effect between hTERT siRNA and Cisplatin in the suppression of HCC progression and indicated that the combination of hTERT-specific siRNA and cisplatin could be an effective therapy for HCC to promote cell cycle controls *via* G2/M and S phases thus inducing apoptosis (102). In another study, stabilized siRNA were tested *in vivo* to demonstrate that the depletion of cyclin E expression in this manner promotes apoptosis in HCC cells and blocks cell proliferation (103). Cyclin E (cyclins E1 and E2) are components of the core cell cycle machinery and are usually co-expressed in proliferating cells (164). Another study using a stabilized siRNA transfection to target CXCL1 induced apoptosis *in vitro* and *in vivo* (104). CXCL1 expression promotes fibrogenesis and angiogenesis HCC (165). In another experiment, the knockdown of PDGFR-ß mRNA by siRNA led to the upregulation of LC3-II and downregulation of p62, indicating that knockdown of PDGFR-ß could activate autophagy and indicating that PDGFR-ß plays a role in the Akt/mTOR and Mek/Erk pathways (105).

A multifunctional polymeric NP, that has progressed to clinical trial stage, has been developed to transport shRNA survivin and VEGF siRNA to trigger apoptosis and inhibit angiogenesis in HCC patients (106). This work was largely based on an earlier study by the same researchers where the polymeric NP was deployed in a mouse to transport VEGF siRNA that demonstrated a pro-apoptotic effect as a result of RNAi silenced VEGF mRNA (107). This demonstrates that VEGF and survivin repression can also influence pro-apoptotic activity, as well anti-angiogenic potential in HCC.

**CRISPR/Cas9**: In one study using a mouse xenograft model, a CRISPR/Cas9 model used Cas9-based genome editing to introduce the prodrug-converting enzyme herpes simplex virus type 1 thymidine kinase (HSV1-tk) into the genomes of HCC cells resulting in the precipitation of apoptosis (108). In another study, CRISPR/Cas9 gene editing was deployed to disrupt the expression of the the programmed death 1 receptor (PD-1) to enhance the activity of CAR T cells. Disruption of PD-1, therefore, enhanced the anti-tumor activity of CAR T cells against HCC (86).

**ASO**: A chemically stabilized ASO was successfully deployed to repress aberrant TERT expression in HCC models to induce apoptosis *in vitro* and *in vivo* (109).

**Aptamer**: A 75 nucleotide long aptamer (GT75) was tested in three HCC cell lines, HepG2, HuH7 and JHH6. When delivered by liposomes. GT75 was able to effectively reduce HCC cell viability and promote apoptosis. No effects on cell cycle were observed and the effect of this aptamer was likely due to the interference with eEF1A activity (110).

**Ribozyme**: A number of *trans*-splicing ribozymes have been tested for RNAi therapeutic applications in HCC, many of which have targeted the elevated expression of telomerase reverse transcriptase (TERT) because of its role in promoting cell survival (112). Examples include an *in vivo* HCC experiment an intra-tumoral injection of an adenovirus encoding for the hTERT-targeting *trans*-splicing ribozyme demonstrated marked regression of tumors that had been subcutaneously inoculated with hTERT-positive liver cancer cells in mice (112). Another RNAi experiment using an hTERT-targeting ribozyme with an miR-122 target site added to its 3’ end effectively promoted cell death because miR-122 is upregulated in HCC and is the most predominant microRNA(miRNA) expressed in liver tissue. Effectively repressing hTERT and miR-122 levels also improved the selectivity of this therapy, as well as reduced hepatotoxicity (113, 114). Other ribozyme targets in HCC targeted (cleaved) mutant TP 53 mRNA and replaced it with normal TP 53 mRNA to promote TP53 expression and induce apoptosis (111), as well as a trans*-*splicing ribozyme-mediated replacement of HCC-associated specific AFP to promote cell suicide (94). Ribozyme mediated suppression of VEGF expression enhanced matrix metalloproteinase 1 (MMP-1)nexpression in annHCC cell line to underline that repressing one oncoprotein can result in stimulating another (67).

**Riboswitch**: In one HCC study a riboswitch that could sense AFP triggered the promotion of YAP induced 14-3-3σ expression to promote degradation (68). A chimeric RNA-binding protein-based killing switch targeting HCC cells deployed a synthetic circuit for cNOT7 that is a deadenylase that can inhibit mRNA translation and promote their degradation. It was demonstrated in this study that the result of this deadenylase triggered apoptosis by switching on bcl-2 and repressing hBAX respectively (95).

## Conclusion

RNA are key molecules involved in all biological pathways. Their history of therapeutic deployment begins with the first antisense oligonucleotide (ASO) drug in 1998, first aptamer drug in 2004, the first RNAi clinical trial in 2010 and the first siRNA drug in 2018. These RNAi drugs were based on the synthetic development of complementary RNA sequences that can bind to mRNA or a protein to repress (silence) expression. Six FDA approved RNA based drugs have been approved from 1998 to 2019 with the majority developed for muscular dystrophy/atrophy (6).

To date, a few clinical trials have been registered for siRNA RNAi based therapy in HCC. These trials include an LNP to target MYC mRNA to repress proliferation, a SNALP to target PLK1 mRNA (79) to retard cell cycle and an LNP to target EPHA2 mRNA to repress angiogenesis. Other trials include a polymer based NP to target PKN3 mRNA to repress vascularization, an LNP to target STMN1 mRNA to repress cell cycle, an LNP to target miR-34, and a polymer based NP to target RRM2 mRNA to repress cell proliferation (31). Another trial deployed a siRNA (miR-34) tumor suppressor to target 20 HCC associated genes, as well as to sensitize the tumor microenvironment to cytotoxic therapy (166). Bearing in mind the first siRNA-based drug was only approved in 2018 the indications are it will be some time before their true potential can be evaluated (6).

Despite the early promise of RNA therapeutics as a magic bullet to modulate aberrant signaling in cancer, this field remains a work-in-progress largely due to the inherent instability of RNA *in vivo*, as well as delivery and host immunogenic issues. RNAi targeting is often problematic because of the cross-talk between genes and some RNAi studies have demonstrated that repressing one oncoprotein can result in stimulating another (67). Nevertheless, RNA therapeutics is now a reality for the prevention of viral diseases (COVID-19) and offers great promise for cancer. Currently, most HCC drugs are based on recombinant proteins or antibodies that target pathologic proteins which are often difficult to reach because of their location in the cell. In addition, these drugs often struggle to differentiate between target protein sub-types, whereas RNA can more easily target specific mRNA or other ncRNA (31). A wide range of novel HCC therapeutics, including RNAi have been developed over the last two decades, however, a cure is not a reality at present (3).

## Author Contributions

KS conceptualized, developed first draft and responded to reviewer edits and comments to develop final draft. SA reviewed and revised drafts, AC reviewed and revised drafts, AK reviewed and revised drafts. All authors contributed to the article and approved the submitted version.

## Conflict of Interest

The authors declare that the research was conducted in the absence of any commercial or financial relationships that could be construed as a potential conflict of interest.

## Publisher’s Note

All claims expressed in this article are solely those of the authors and do not necessarily represent those of their affiliated organizations, or those of the publisher, the editors and the reviewers. Any product that may be evaluated in this article, or claim that may be made by its manufacturer, is not guaranteed or endorsed by the publisher.
